# Structure-Guided Discovery Reveals Recurrent Bioactive Peptide Architectures Across Coleoptera

**DOI:** 10.3390/ijms27167489

**Published:** 2026-08-21

**Authors:** Thaís Caroline Gonçalves, João Alfredo Teodoro, Danilo T. Amaral

**Affiliations:** Centro de Ciências Naturais e Humanas, Universidade Federal do ABC (UFABC), Santo André 09210-580, SP, Brazil; thais.goncalves@ufabc.edu.br (T.C.G.); joao.teodoro@ufabc.edu.br (J.A.T.)

**Keywords:** AlphaFold, bioactive peptides, Coleoptera, machine learning, structural bioinformatics

## Abstract

Bioactive peptides are an important source of therapeutic molecules and molecular scaffolds involved in defense, signaling, and immune regulation. Despite the extraordinary diversity of Coleoptera, the structural landscape of beetle-derived bioactive peptides remains largely unexplored, limiting our understanding of their evolutionary diversity and biotechnological potential. Here, we performed a large-scale structural survey of predicted toxin-like peptide scaffolds across publicly available Coleoptera transcriptomes by integrating transcriptome mining, peptide maturation prediction, physicochemical characterization, AlphaFold 3 structural modeling, structural similarity analyses, and interpretable machine learning. We identified 291 candidate peptides, of which 155 contained canonical signal peptides and 273 produced mature peptides within the expected size range of known bioactive peptides. Structural analyses revealed that, despite extensive sequence diversity, many candidates were organized into a comparatively restricted repertoire of compact cysteine-rich architectures, indicating that structural similarity is retained across peptides exhibiting substantial primary-sequence variation. Comparative structural analyses further identified recurrent protein architectures shared across multiple beetle lineages, while machine learning prioritization integrated structural and biochemical descriptors to identify high-confidence candidates for future functional characterization. These analyses establish the first structural atlas of predicted toxin-like peptides across Coleoptera and demonstrate that structure-guided transcriptome mining provides a powerful framework for uncovering recurrent bioactive peptide scaffolds that would remain largely undetected using sequence-based approaches alone. Beyond expanding our understanding of peptide evolution in beetles, this resource is a foundation for future structural, functional, and biotechnological exploration of bioactive peptides in underexplored animal groups.

## 1. Introduction

Bioactive peptides constitute one of the most diverse classes of biologically active molecules in nature, participating in a wide range of physiological processes including defense, signaling, immune regulation, enzyme inhibition, and interspecific interactions. Their remarkable structural diversity, combined with high specificity and favorable pharmacological properties, has made these molecules valuable sources of therapeutic agents, agricultural bioproducts, and molecular probes [1,2,3]. Among these molecules, small secreted cysteine-rich peptides are particularly notable because compact disulfide-stabilized architectures frequently preserve structural integrity while supporting extensive functional diversification [4,5,6].

The discovery of bioactive peptides has focused predominantly on a relatively small number of well-characterized venomous organisms, including snakes, spiders, cone snails, scorpions, and marine invertebrates [6,7]. However, accumulating evidence suggests that structurally related peptide scaffolds extend far beyond these traditional groups and may perform diverse biological functions unrelated to venom, including antimicrobial activity, immune modulation, and cellular signaling [2,8,9]. Consequently, taxonomically diverse yet poorly explored groups may harbor a vast and largely unrecognized diversity of bioactive peptide scaffolds.

Coleoptera represents an outstanding example of this knowledge gap. As the largest animal order, comprising nearly one-quarter of all described animal species, beetles occupy virtually every terrestrial ecosystem and exhibit extraordinary ecological and evolutionary diversification. Despite this remarkable diversity, comparatively little is known about the repertoire, structural organization, and evolutionary relationships of their small secreted peptides. Most available studies have focused on individual antimicrobial peptides or isolated toxin-like molecules, leaving the broader structural diversity of beetle-derived bioactive peptides largely unexplored [3,8]. This disparity raises an important question: does the apparent scarcity of beetle bioactive peptides reflect genuine biological rarity, or simply limited exploration?

Recent advances in artificial intelligence and structural bioinformatics have fundamentally transformed protein discovery. High-accuracy structure prediction methods, particularly AlphaFold 3, together with large-scale structural comparison tools such as Foldseek, now enable protein relationships to be explored beyond primary sequence conservation [10,11]. Increasingly, protein structure is recognized as a more conserved and functionally informative descriptor than sequence alone, allowing the identification of structurally related protein architectures that would otherwise remain undetected [12,13]. These developments have shifted peptide discovery from predominantly sequence-based approaches toward integrative structure-guided strategies capable of exploring previously inaccessible regions of protein structural space.

Here, we present the first large-scale structural survey of predicted toxin-like peptide scaffolds across Coleoptera. By integrating transcriptome mining, peptide maturation prediction, physicochemical characterization, AlphaFold 3 structural modeling, structural similarity analyses, and interpretable machine learning, we generated a comprehensive structural atlas of bioactive peptide candidates spanning diverse beetle lineages. Rather than focusing solely on peptide identification, our objective was to investigate how structural information organizes peptide diversity across Coleoptera and whether recurrent protein architectures emerge despite extensive sequence divergence. Together, our results demonstrate that beetle transcriptomes harbor a previously underappreciated repertoire of recurrent and structurally similar bioactive peptide scaffolds and establish a scalable framework for structure-guided peptide discovery in underexplored animal groups.

## 2. Results

### 2.1. Large-Scale Discovery of Toxin-like Peptide Candidates Across Coleoptera

The analyses identified 291 short peptide candidates exhibiting characteristics compatible with toxin-like molecules, providing one of the first large-scale overviews of putative toxin-like peptides across the order (Table 1). These candidates were recovered from multiple beetle lineages, indicating that short secreted peptides are broadly distributed throughout Coleoptera rather than being restricted to a small number of taxa.

Among the identified candidates, 155 peptides (53.3%) were predicted to contain N-terminal signal peptides, supporting their potential secretion through the classical secretory pathway (Table 1). Because extracellular secretion represents a fundamental requirement for most peptide toxins and other bioactive peptides [14,15], this result substantially enriches the candidate dataset for molecules with potential biological activity. The remaining candidates lacking predicted signal peptides were retained for subsequent analyses because they frequently exhibited additional characteristics consistent with bioactive peptides, including appropriate mature peptide length, cysteine-rich architectures, and structurally stable AlphaFold models. Their inclusion reflects the exploratory nature of the discovery pipeline and does not imply that these molecules are classically secreted peptides.

**Figure 1:**
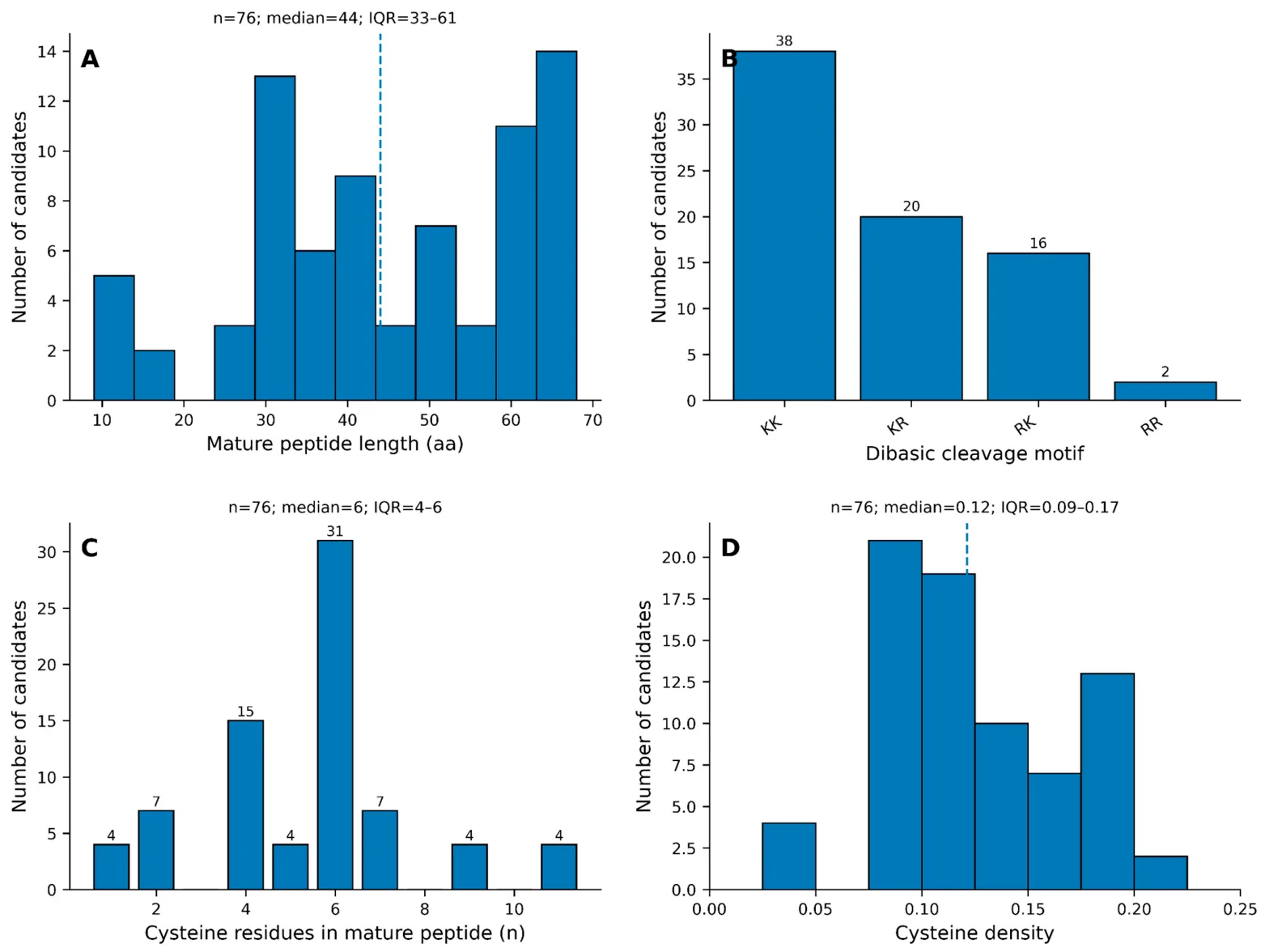
(**A**) Distribution of inferred mature peptide lengths of the 76 high-confidence Coleoptera. (**B**) Distribution of dibasic cleavage motifs identified in the precursor sequences downstream of the predicted signal peptide and used as evidence of proteolytic processing. (**C**) Number of cysteine residues in the inferred mature peptide sequences. (**D**) Cysteine density of the inferred mature peptide sequences. Dashed lines in the continuous-variable panels indicate the median, with the median and interquartile range (IQR) reported in each panel.

To assess their maturation potential, precursor sequences were computationally processed using predicted signal peptide cleavage sites together with canonical proteolytic processing rules. Following maturation inference, 273 candidates (93.8%) generated mature peptides within the expected size range of 8–80 amino acids, consistent with the size distribution commonly reported for bioactive toxin-like peptides (Table 1) [8]. Only 18 sequences (6.2%) produced mature peptides outside this interval and were therefore considered lower-confidence candidates. Proteolytic processing signals were also frequently observed. A total of 127 candidates (43.6%) contained canonical dibasic cleavage motifs (e.g., KR, RR, and KK) downstream of the signal peptide, supporting precursor architectures compatible with regulated peptide maturation [1,16]. Following maturation inference, cysteine content was recalculated exclusively from the inferred mature peptide region, thereby excluding cysteine residues located within signal peptides or removed propeptide segments (Figure 1).

### 2.2. Physicochemical Landscape of Coleoptera Toxin-like Peptides

**Figure 2:**
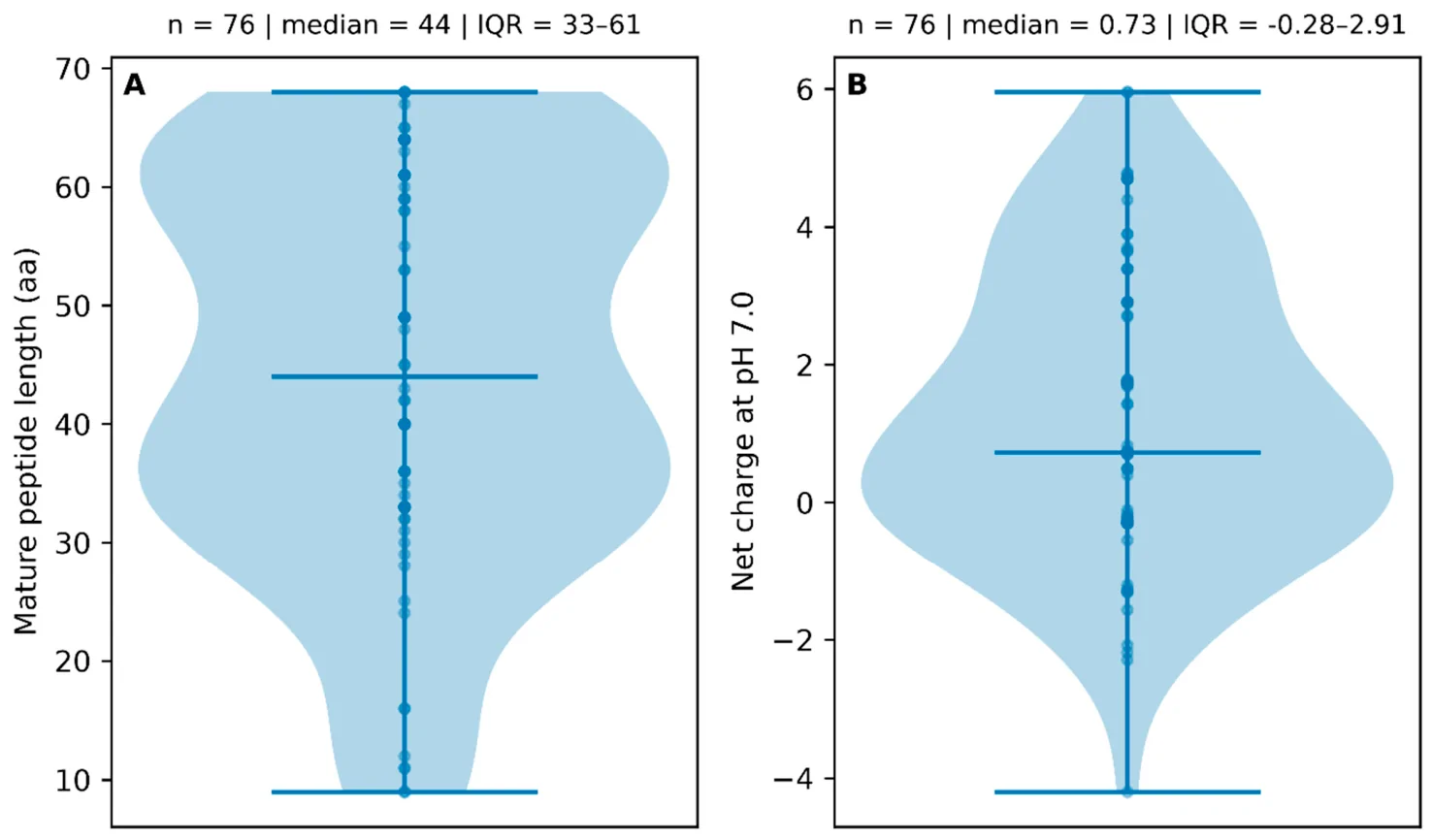
Physicochemical properties of the 76 high-confidence mature Coleoptera toxin-like peptide candidates retained after the revised filtering and maturation workflow. Violin plots summarizing the distribution of key sequence-derived features across candidate peptides identified in Coleoptera transcriptomes. (**A**) Peptide length distribution. (**B**) Net charge distribution calculated at pH 7. Each panel includes the median, interquartile range, and summary statistics.

We analyzed the physicochemical properties of the high-confidence mature peptide sequences, including peptide length, molecular weight, net charge, isoelectric point, hydrophobicity, aliphatic index, and mature-peptide cysteine content (Figure 2; Tables S1 and S3). Together, these descriptors provide a comprehensive overview of the biochemical landscape occupied by the identified candidates and offer important insights into their potential structural and functional properties. The retained mature peptides showed substantial variation in length, net charge, and cysteine content (Figure 2). Net charge was calculated at pH 7.0, allowing candidates to be consistently classified along a continuum from negatively to positively charged peptides rather than simply according to the presence of basic or acidic residues.

The predicted mature peptides exhibited substantial variability in sequence length and molecular weight, reflecting the broad diversity expected among naturally occurring bioactive peptides while remaining within the size range commonly reported for toxin-like molecules (Figure 2A; Table S1). Similarly, predicted net charge, calculated at physiological pH (pH 7.0), ranged from negative to positive values (Figure 2B), indicating substantial electrostatic diversity among the mature peptide candidates. Such variation is characteristic of bioactive peptide families that have evolved to recognize distinct molecular targets while maintaining compact extracellular architectures [8]. One of the most distinctive characteristics of the retained mature-peptide dataset was the prevalence of cysteine-rich sequences (Figure 1C,D). Because cysteine counts were recalculated after removal of predicted signal and propeptide regions, these values directly describe the cysteine frameworks of the peptide sequences subjected to physicochemical and structural analyses.

Additional physicochemical descriptors further supported the diversity of the identified candidates. Predicted isoelectric point, hydrophobicity, and aliphatic index varied substantially among peptides (Table S1), suggesting differences in folding behavior, molecular stability, and interaction potential with proteins or membrane-associated targets. Despite this variability, the overall physicochemical distributions remained consistent with those reported for structurally constrained secreted peptides, which encompass a wide range of biological activities beyond classical toxin function, including antimicrobial defense, immune modulation, signaling, and enzyme inhibition [1,2].

One of the most distinctive characteristics of the dataset was the abundance of cysteine-rich peptides (Figure 1C,D). Many candidates contained multiple cysteine residues arranged in patterns compatible with disulfide bond formation, a defining feature of numerous extracellular bioactive peptide families. Disulfide bonds play a central role in stabilizing compact three-dimensional conformations, increasing resistance to proteolytic degradation while preserving structural integrity and molecular specificity [4,5,6]. The widespread occurrence of cysteine-rich architectures therefore suggests that structural stabilization has been a major determinant of peptide evolution within the identified candidates. These physicochemical analyses indicate that the predicted peptides occupy a biochemical space highly consistent with structurally constrained bioactive peptides. Although the candidates display considerable diversity in their primary sequence composition and physicochemical properties, they also share conserved molecular characteristics commonly associated with secreted extracellular peptides. This combination of diversity and conserved biochemical signatures suggests that common structural frameworks may underlie these molecules despite extensive sequence variation, providing the rationale for the structural analyses presented in the following section.

### 2.3. Structural Landscape of Coleoptera Toxin-like Peptides

**Figure 3:**
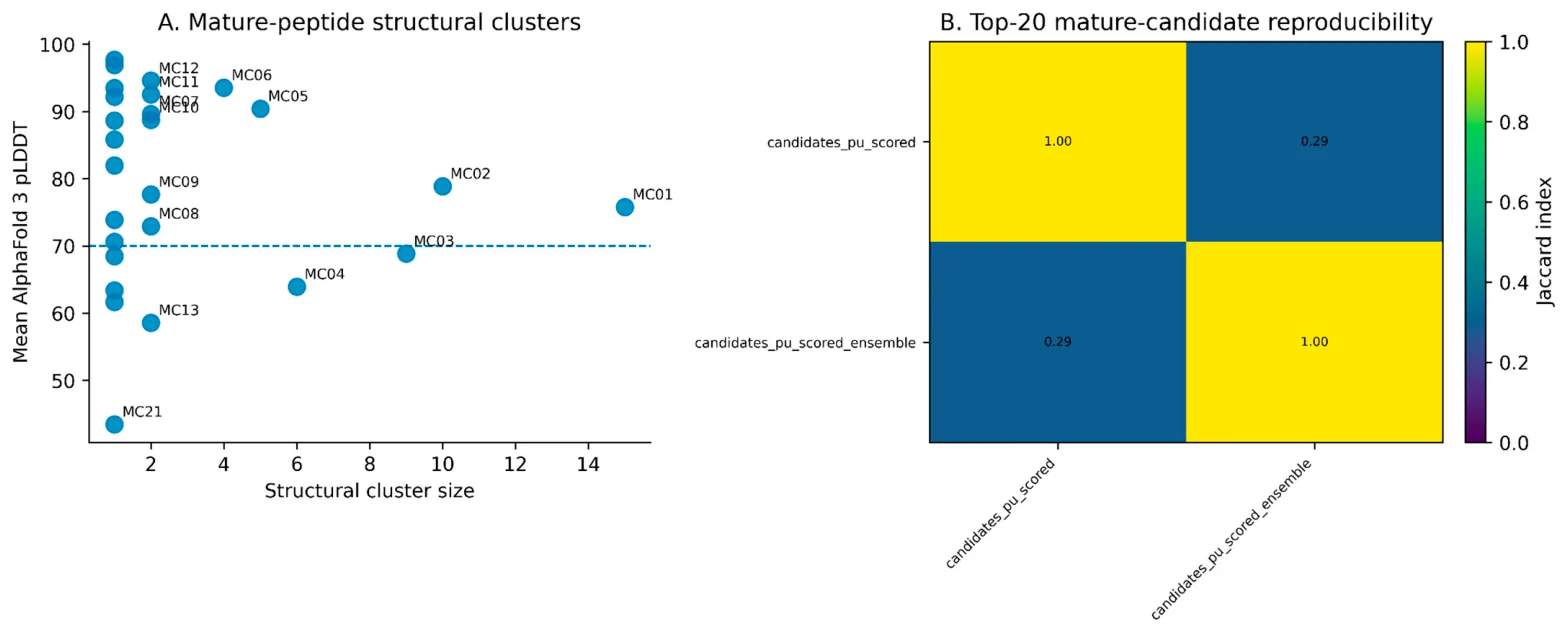
Structural clustering and reproducibility of mature candidate peptides. (**A**) Relationship between cluster size and mean structural confidence (pLDDT), showing that recurrent folds are supported by consistently high-confidence predictions. (**B**) Pairwise Jaccard overlap of the 20 top-ranked candidates across independent runs, indicating reproducible identification of a core subset of peptides.

To investigate the structural diversity of the identified candidates, three-dimensional models were generated using AlphaFold 3 and subsequently characterized through multiple complementary structural descriptors, including predicted Local Distance Difference Test (pLDDT), secondary structure composition, radius of gyration (Rg), solvent-accessible surface area (SASA), and disulfide bond content (Figure 3; Table S2).

The predicted structures exhibited high confidence, with most candidates presenting mean pLDDT values compatible with reliable backbone prediction (Figure 3; Table S2). As expected for short secreted peptides, regions displaying lower confidence were predominantly restricted to terminal residues and exposed loop segments, whereas the structural cores generally exhibited consistently high confidence scores. These observations indicate that the predicted models provide a suitable framework for comparative structural analyses. Despite considerable sequence diversity, the candidate peptides shared several common structural characteristics. Most models adopted compact globular conformations composed of short α-helices, β-strands, and connecting loops arranged in diverse combinations (Figure 3). Radius of gyration and solvent-accessible surface area varied according to peptide length, although the majority of candidates remained within the expected range for small extracellular peptides.

A prominent feature of the dataset was the high frequency of cysteine-rich structures. In many candidates, cysteine residues occupied conserved spatial positions compatible with the formation of stabilizing disulfide bonds, generating compact architectures characteristic of structurally constrained peptide families. Such structural stabilization is widely recognized as a defining characteristic of numerous toxin-like peptides, contributing to both conformational stability and resistance to proteolytic degradation [5].

Individual structures displayed substantial topological variation, however, the overall structural landscape revealed a relatively limited repertoire of recurrent architectural solutions. This observation indicates that diverse primary sequences can adopt similar compact three-dimensional organizations that preserve structural stability while potentially accommodating distinct biological functions. These results establish the first large-scale structural overview of predicted toxin-like peptides across Coleoptera and provide the foundation for subsequent analyses of structural similarity and candidate prioritization.

Interestingly, structural diversity appeared considerably lower than the observed sequence diversity. While mature peptide sequences varied substantially in amino acid composition, length, and physicochemical properties, many candidates grouped into a relatively limited repertoire of compact three-dimensional organizations. This pattern suggests that structural constraints strongly influence the organization of toxin-like peptide diversity, allowing similar molecular architectures to be retained despite extensive primary-sequence variation. These observations motivated a comprehensive structural similarity analysis to determine whether the predicted peptides could be organized into recurrent structural families across Coleoptera.

### 2.4. Structural Similarity Reveals Recurrent Peptide Architectures

Unlike sequence-based comparisons, structural similarity analysis enables the identification of conserved protein architectures even among peptides sharing little primary sequence identity. Structural clustering revealed that the 291 predicted peptides could be organized into a limited number of recurrent structural groups, despite exhibiting substantial sequence variability. Many candidates repeatedly adopted similar three-dimensional organizations characterized by compact folds stabilized by short secondary-structure elements and disulfide-rich cores. These results indicate that structurally constrained peptide architectures recur across multiple beetle lineages, revealing a limited set of structural organizations among the predicted toxin-like peptides analyzed here. The analysis of the 76 high-confidence mature peptide structures resulted in 26 Foldseek-derived structural clusters, encompassing both recurrent architectures and low-representation structural groups (Figure S1 and Table S4). The largest Foldseek-derived clusters contained recurrent architectures represented by dozens of candidates, with the predominant clusters displaying consistent Pfam annotations and generally high structural confidence (Figure S1). The highest-scoring PU-learning representative from each cluster was retained to facilitate structural interpretation and downstream visualization.

Sequence-based comparisons provided an independent layer of evidence for the structural classifications. BLASTP searches of the representative mature peptides against UniProtKB/Swiss-Prot recovered at least one sequence-similarity hit for 25 of the 26 representatives. Several clusters showed substantial similarity to previously characterized bioactive peptide families, including defensin-like peptides and protease-inhibitor architectures, whereas other representatives showed only moderate or limited sequence support (Table S4).

Several structural clusters included peptides originating from different Coleoptera families, indicating that similar protein architectures are distributed across multiple taxonomic lineages. However, structural similarity alone does not establish whether these architectures arose independently, were retained from shared ancestral scaffolds, or emerged through parallel evolutionary processes. In addition, some structural groups were represented by only a few candidates, highlighting the presence of potentially lineage-restricted architectures that may correspond to previously unrecognized structural scaffolds. Together, the recurrent and less frequently represented folds reveal a structural landscape characterized by both broadly distributed and taxonomically restricted architectures, expanding the currently recognized diversity of small extracellular peptide scaffolds in Coleoptera. To provide an independent functional context for these structural relationships, representative sequences were compared against curated UniProtKB/Swiss-Prot proteins and associated Gene Ontology annotations were examined (Table S4).

Structural similarity analyses demonstrate that the remarkable sequence diversity observed among the candidate peptides collapses into a comparatively restricted structural landscape. This reduction in structural complexity suggests that recurrent protein fold conservation represents a major organizing feature of toxin-like peptide diversity in Coleoptera and supports the construction of the first structural atlas of these molecules across the order.

### 2.5. Structural Features Enable the Prioritization of High-Confidence Toxin-like Peptides

**Figure 4:**
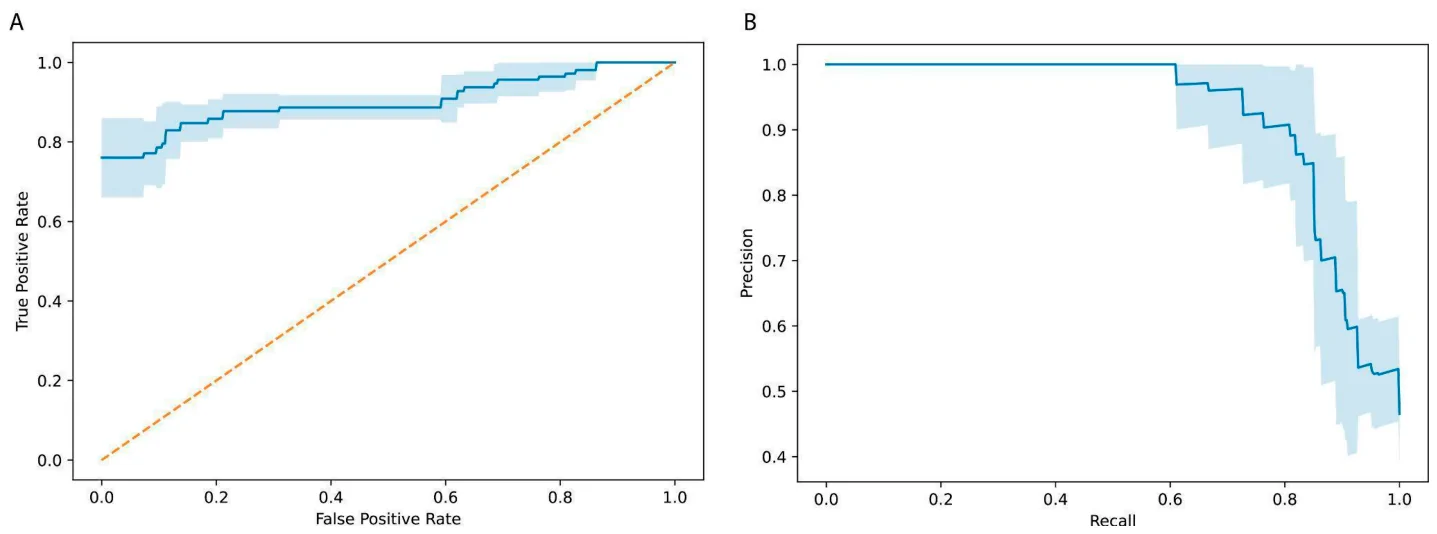

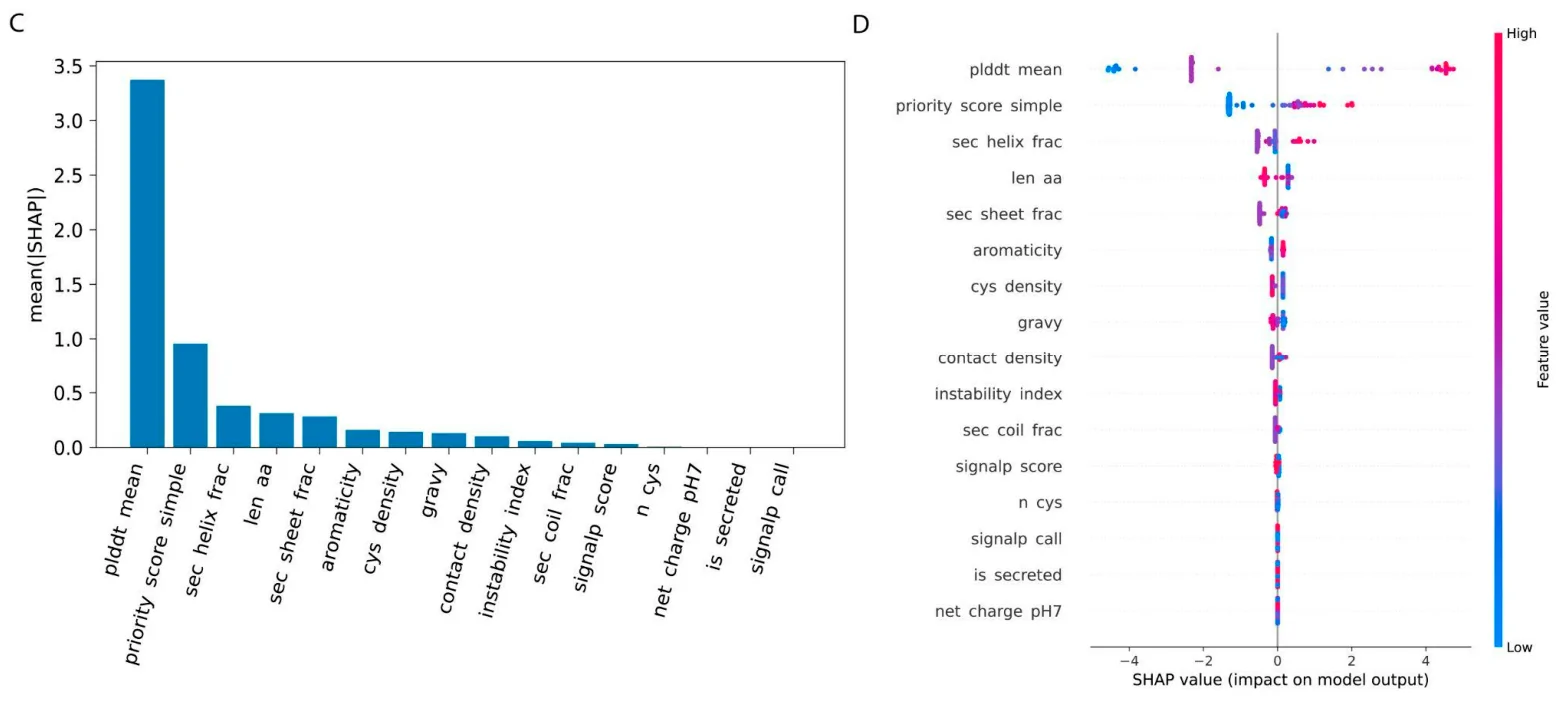
Performance of the PU-learning framework showing (**A**) model evaluation metrics, (**B**) ROC and Precision–Recall curves, (**C**) SHAP feature importance, and (**D**) prioritization of high-confidence toxin-like peptide candidates.

Following structural characterization, we integrated sequence-derived, physicochemical, and structural descriptors into a PU learning framework to prioritize candidates with the highest probability of representing functional toxin-like peptides (Figure 4). Unlike conventional binary classifiers, PU-learning is particularly suitable for biological discovery problems where experimentally validated positive examples are available, whereas true negative examples remain largely unknown.

Model performance indicated that the integration of sequence-derived, physicochemical, and structural descriptors supported effective candidate discrimination. Structural variables, including AlphaFold-derived confidence scores and three-dimensional descriptors, contributed to candidate prioritization, indicating that protein architecture provides complementary information beyond primary sequence composition. Feature importance analysis using SHAP revealed that structural characteristics consistently ranked among the most informative predictors driving candidate prioritization (Figure 4). Rather than relying on a single descriptor, the model integrated multiple independent sources of evidence, including secretion signals, peptide maturation, physicochemical properties, and structural features, allowing robust discrimination of high-confidence candidates.

The resulting prioritization identified a subset of top-ranked peptides that simultaneously exhibited canonical secretion signals, mature peptide lengths consistent with known toxin families, structurally stable AlphaFold models, and physicochemical properties compatible with extracellular bioactive peptides. These candidates represent the most promising molecules for future experimental validation and functional characterization. The prioritization framework demonstrates that combining structural information with interpretable machine learning presents an effective strategy for reducing the large search space generated by transcriptome mining while preserving molecular diversity. Rather than replacing biological interpretation, the machine learning model functions as a complementary decision-support tool that systematically integrates diverse lines of evidence into a unified confidence ranking.

We compiled the 76 highest-confidence candidates integrating secretion prediction, physicochemical properties, structural confidence, structural clustering, and Positive-Unlabeled learning scores (Table 2). Most top-ranked peptides displayed high AlphaFold 3 confidence (mean pLDDT > 85), multiple cysteine residues compatible with disulfide-rich architectures, and were consistently recovered across independent ensemble runs. Several candidates belonged to recurrent structural clusters shared across different distant Coleoptera lineages, whereas others displayed less frequently observed structural architectures, highlighting both broadly distributed and potentially lineage-restricted peptide scaffolds for future biochemical characterization.

**Figure 5:**
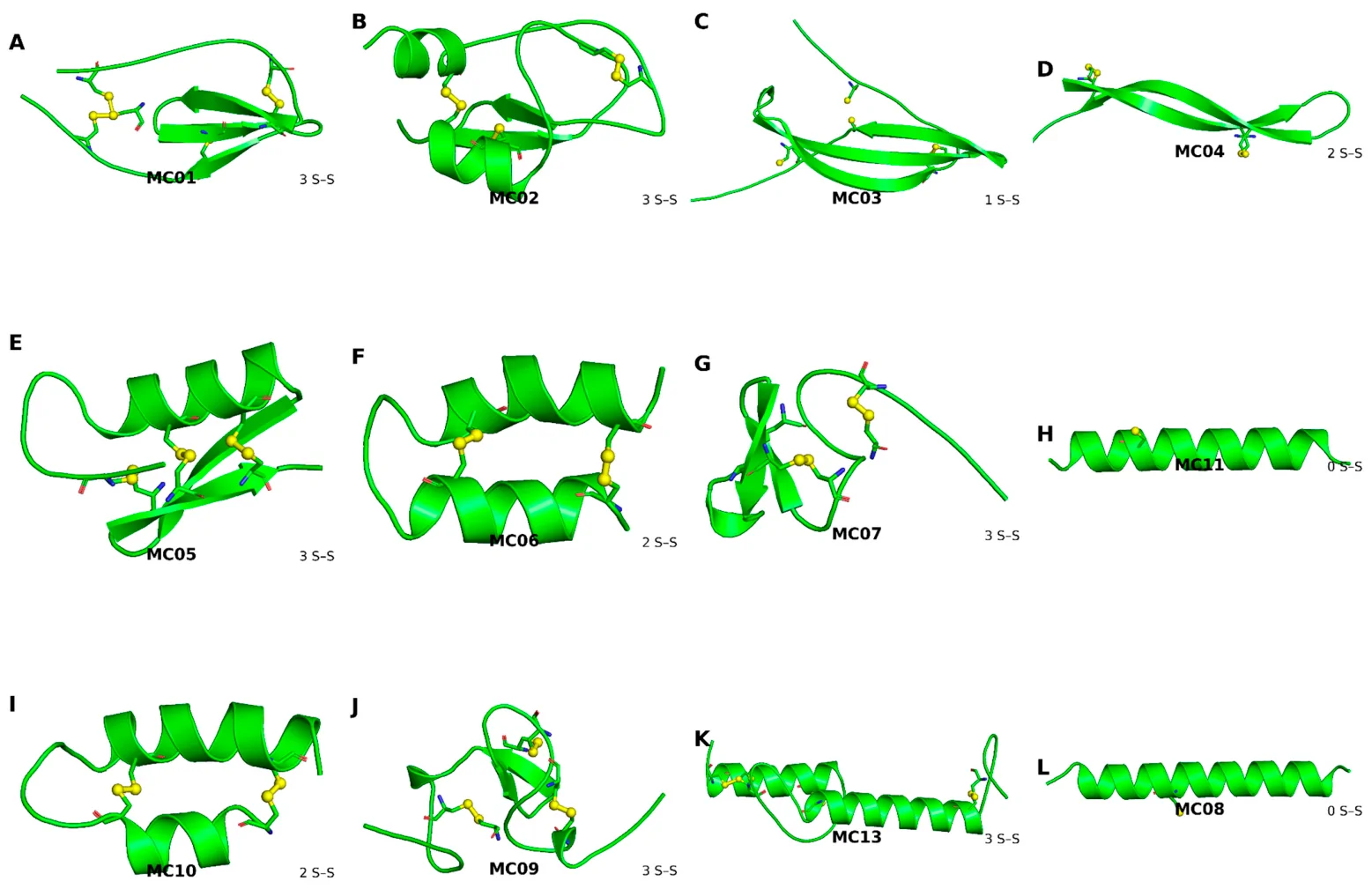
Representative AlphaFold 3 structures of mature toxin-like peptide clusters across Coleoptera. Twelve representative structural clusters obtained from the 76 high-confidence mature peptide candidates are shown (**A**–**L**). Predicted disulfide bonds between cysteine residues are indicated by S-S connections. Structures were modeled exclusively from inferred mature peptide sequences and clustered using Foldseek. MC08 showed no close structural counterpart in PDB or AlphaFold/Swiss-Prot. The complete details are provided in Supplementary Figure S1 and Table S4.

**Figure 6:**
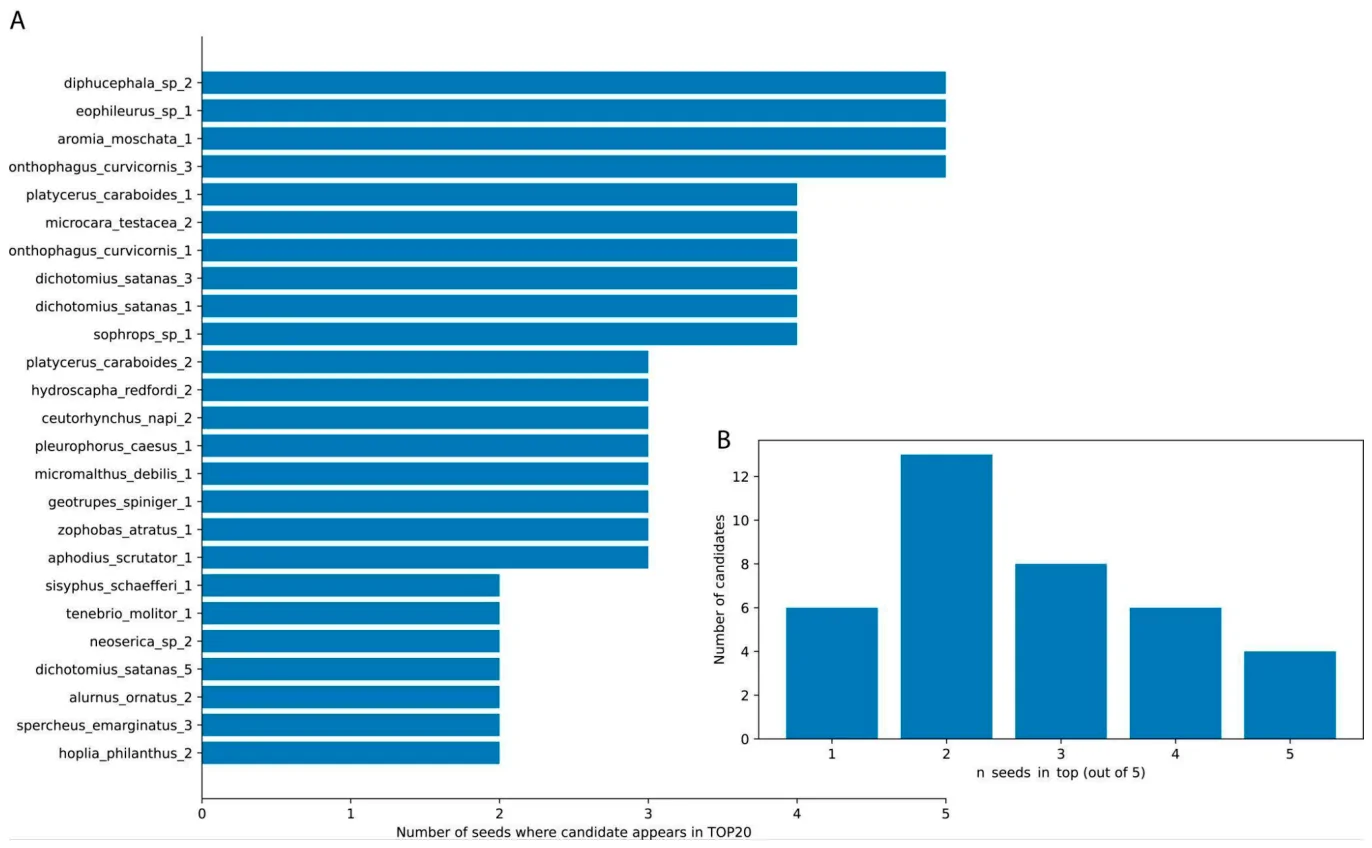
Stability of top-ranked candidates across independent runs. (**A**) Number of seeds in which each candidate appears among the top 20, highlighting highly recurrent peptides. (**B**) Distribution of candidate recurrence across seeds, showing the presence of a stable subset consistently identified by the model.

Representative structures from the major Foldseek-derived clusters were visually compared (Figure 5). Despite originating from taxonomically distant beetle species, several candidates shared similar three-dimensional architectures, whereas others represented distinct structural scaffolds, illustrating the structural diversity recovered by the prioritization pipeline. The stability of candidate prioritization was further evaluated across the five independently trained PU-learning models (Figure 6). Several candidates repeatedly appeared among the 20 top-ranked predictions across different random seeds (Figure 6A), revealing a stable core of highly recurrent candidates. The overall recurrence distribution further showed that a subset of candidates was consistently recovered across multiple independent runs (Figure 6B), supporting the robustness of the prioritization against stochastic variation in model training.

### 2.6. A Structural Atlas of Coleoptera Toxin-like Peptides

The integration of transcriptome mining, physicochemical characterization, structural prediction, structural similarity analyses, and machine learning prioritization resulted in the first large-scale structural atlas of predicted toxin-like peptides across Coleoptera (Figure 5; Table S3). Rather than representing an isolated collection of peptide sequences, the resulting dataset is a comprehensive resource linking sequence, biochemical properties, three-dimensional structure, structural similarity, and prioritization scores for each candidate.

The atlas encompasses peptides distributed across multiple beetle lineages and captures a broad spectrum of structural organizations, ranging from recurrent compact architectures to structural variants detected in restricted subsets of the analyzed taxa. While recurrent structural folds dominated the dataset, several candidates occupied unique regions of the structural landscape, representing potentially novel peptide scaffolds that currently lack close structural counterparts among the remaining Coleoptera candidates according to Foldseek clustering (Figure 5). Unlike the highly populated structural clusters, these low-representation clusters contained single or very few mature peptides displaying unique structural organizations. These candidates therefore represent the most promising putatively novel structural scaffolds identified in the present survey and constitute attractive targets for future experimental characterization. These molecules constitute promising targets for future structural, biochemical, and pharmacological investigations.

External structural comparisons revealed that most representative clusters exhibited detectable relationships to previously characterized structural architectures, ranging from close to distant similarities (Figure S1; Table S4). To avoid inferring structural novelty solely from low cluster representation, selected candidates lacking close counterparts in the initial search were subjected to additional sensitive Foldseek searches against both the PDB and AlphaFold/Swiss-Prot structural collections. These analyses recovered close structural counterparts for MC11 and MC25, whereas MC08 remained without a detectable close structural counterpart in either database. MC08 is therefore highlighted in Figure 5 and Figure S1 as a candidate structural architecture lacking a close counterpart under the search conditions employed, rather than being classified as a definitively novel fold.

The prioritization framework further facilitates exploration of this structural diversity by identifying candidates that simultaneously exhibit canonical secretion signals, appropriate maturation patterns, structurally reliable models, and favorable physicochemical characteristics. Rather than limiting future studies to a small number of highly similar peptides, the atlas preserves the observed structural diversity while providing a rational strategy for selecting representative candidates from distinct structural groups. This resource establishes a foundation for future studies investigating the evolution, molecular function, and biotechnological potential of beetle-derived toxin-like peptides. By integrating complementary computational approaches into a unified structural framework, the atlas considerably expands the currently available repertoire of predicted peptide structures from Coleoptera.

## 3. Discussion

### 3.1. Coleoptera Harbor an Overlooked Structural Diversity of Toxin-like Peptides

To our knowledge, this is the first large-scale structural survey of predicted toxin-like peptides across Coleoptera, revealing that beetle transcriptomes harbor a considerably broader repertoire of secreted and structurally constrained peptides than previously recognized. Although beetles comprise nearly one-quarter of all described animal species, their peptide diversity has received remarkably limited attention compared with classical venomous organisms such as snakes, spiders, cone snails, scorpions, and marine cnidarians [5,17]. Consequently, the structural diversity of beetle-derived bioactive peptides has remained largely unexplored.

Our analyses indicate that this apparent lack of diversity likely reflects a historical sampling bias rather than a genuine biological absence of toxin-like peptides. By integrating transcriptome mining with structural prediction and comparative analyses, we identified hundreds of candidates displaying the hallmark characteristics of secreted bioactive peptides, including canonical signal peptides, conserved maturation features, cysteine-rich architectures, and compact three-dimensional structures. Collectively, these observations suggest that beetles represent an overlooked reservoir of structurally diverse peptide scaffolds with considerable evolutionary and biotechnological relevance.

Importantly, our findings should not be interpreted as evidence that all identified candidates function as toxins. Instead, the observed structural characteristics indicate that these peptides occupy a region of protein structural space commonly associated with extracellular bioactive molecules. This distinction is particularly important because similar structural frameworks may support diverse biological functions, including antimicrobial activity, immune regulation, signaling, enzyme inhibition, and defense. Thus, the structural atlas presented here substantially expands the repertoire of candidate peptide scaffolds available for future functional characterization while highlighting the value of transcriptome-scale structural exploration in taxonomically underrepresented animal groups.

### 3.2. Recurrent Structural Architectures Organize Coleoptera Bioactive Peptide Scaffolds

One of the most striking observations of this study is that the extensive sequence diversity identified across Coleoptera is organized into a comparatively restricted repertoire of three-dimensional protein architectures. Although the predicted mature peptides differed substantially in amino acid composition, length, and physicochemical properties, structural analyses consistently revealed recurrent compact folds stabilized by similar secondary-structure arrangements and cysteine-rich cores. This apparent reduction in structural complexity indicates that protein architecture is more strongly retained than primary sequence across the analyzed candidates. Similar observations have emerged from large-scale structural analyses showing that related or functionally analogous protein folds can remain detectable despite extensive primary-sequence variation, reinforcing the concept that structural space is more constrained than sequence space [11,13].

Recurrent structural architectures can result from different evolutionary processes. Similar folds may arise independently through convergent evolution, may develop in related lineages under comparable selective pressures through parallel evolution, or may be retained and subsequently diversified from a shared ancestral scaffold. Structural similarity alone cannot distinguish among these alternatives. Although recurrent architectures have been extensively documented among venom and bioactive peptide families [5,7,9,18,19], establishing their evolutionary origin requires explicit phylogenetic analyses, including gene-family reconstruction, ancestral-state inference, and evaluation of taxonomic distribution.

In the present study, the occurrence of similar three-dimensional architectures among candidates from different Coleoptera families demonstrates broad structural recurrence but does not, by itself, establish independent evolutionary origins. Therefore, these patterns should not be interpreted as direct evidence of convergent evolution. Shared ancestry, parallel evolution, and independent structural convergence remain plausible and non-mutually exclusive explanations. Our results provide a structural framework for identifying these recurrent architectures, while future phylogenetic comparative analyses will be required to determine the evolutionary processes responsible for their distribution.

The structural gallery shown in Figure 5 illustrates that multiple high-confidence candidates share recurrent three-dimensional architectures despite substantial differences in sequence composition and taxonomic origin. This observation supports the conclusion that structural similarity can exceed detectable primary-sequence similarity among the analyzed Coleoptera toxin-like peptides. It also reinforces the value of integrating structure-based analyses with machine learning for large-scale peptide discovery, independently of the evolutionary mechanism responsible for the observed structural recurrence.

The predominance of cysteine-rich candidates provides a mechanistic explanation for part of this structural recurrence. Disulfide bonds represent an efficient solution for stabilizing small extracellular peptides, increasing resistance to proteolytic degradation while maintaining well-defined conformations compatible with receptor recognition and molecular specificity [4,5]. Conserved cysteine arrangements and oxidative folding constraints can preserve protein architecture even after extensive sequence variation [6]. Likewise, computational and structural studies have highlighted that disulfide-rich scaffolds provide adaptable frameworks capable of supporting multiple biological activities while maintaining structural robustness [12].

Importantly, shared structural architecture should not be interpreted as evidence of functional equivalence. Similar protein folds can support antimicrobial defense, immune modulation, signaling, enzyme inhibition, or toxin activity. Structurally related peptide families may undergo extensive functional diversification, with comparable scaffolds recruited for distinct physiological processes [2,3]. Rather than predicting a single biological role, the architectures identified here should therefore be interpreted as versatile candidate scaffolds whose functions remain to be experimentally established.

Taken together, these results support the view that Coleoptera toxin-like peptide diversity is organized around a relatively restricted number of recurrent protein architectures. This observation shifts the emphasis from primary-sequence similarity toward structural similarity as a complementary framework for peptide discovery. However, the present analyses characterize structural recurrence rather than its evolutionary origin, which remains an important question for future phylogenetic and comparative investigations.

### 3.3. Structural Information Enhances Computational Discovery and Prioritization of Bioactive Peptides

A major advantage of the framework presented here lies in the integration of structural information into the computational discovery process. Traditional peptide mining strategies rely predominantly on sequence similarity, conserved motifs, or physicochemical descriptors, which often fail to detect highly divergent peptide families sharing common structural architectures. By incorporating AlphaFold-derived models, structural descriptors, and Foldseek-based comparisons (Figure 3 and Figure 4), our approach substantially expands the search space beyond primary sequence conservation, allowing the identification of candidates that would likely remain undetected using conventional homology-based strategies. Recent advances in structure prediction have similarly demonstrated that structural information is becoming an essential component of protein annotation and functional inference, particularly for rapidly evolving protein families [11,12].

The explainable machine learning framework further demonstrated that structural descriptors contributed substantially to candidate prioritization. Rather than depending on a single predictive feature, the model integrated complementary sources of evidence, including secretion signals, precursor maturation, physicochemical properties, and three-dimensional structural characteristics. Feature attribution analyses based on SHAP confirmed that several structural variables ranked among the most influential predictors driving model decisions (Figure 4), highlighting that structural information captures biologically meaningful patterns not readily identifiable from sequence alone [20,21]. This observation agrees with recent studies showing that combining structural modeling with interpretable machine learning considerably improves the discovery of bioactive peptides compared with sequence-only approaches [12,22].

An additional strength of our strategy is the use of PU learning. Unlike conventional supervised classification, PU-learning is specifically designed for discovery problems in which experimentally validated positive examples are available but reliable negative datasets are largely absent. This characteristic makes the approach particularly suitable for peptide discovery, where the absence of functional annotation cannot be interpreted as evidence of inactivity. Consequently, the prioritization framework shows probabilistic estimates of confidence rather than definitive functional assignments, reducing the risk of introducing biases associated with artificially generated negative datasets [23]. Moreover, because peptide discovery typically involves highly imbalanced datasets, the combined interpretation of ROC and Precision-Recall curves (Figure 4 displays a more informative assessment of classifier performance than either metric alone) [24].

The computational framework should not be viewed as a substitute for experimental validation but rather as an efficient strategy for reducing an otherwise overwhelming search space. The structural atlas generated in this study (Table S3) contains hundreds of candidates displaying diverse architectures and biochemical characteristics, making exhaustive functional characterization impractical. By integrating structural prediction, structural similarity, and explainable machine learning into a unified prioritization workflow, our approach enables the rational selection of representative candidates spanning distinct structural groups, thereby maximizing structural diversity while minimizing experimental effort. These findings reinforce the growing paradigm that protein structure provides a more robust framework than sequence similarity alone for guiding the discovery of novel bioactive peptides. As high-accuracy structural prediction and comparative structural bioinformatics continue to advance, integrative approaches such as the one presented here are expected to play an increasingly central role in large-scale peptide discovery and functional annotation across underexplored taxa [11,12,13].

### 3.4. Biological Significance and Biotechnological Opportunities of the Structural Atlas

Beyond expanding our understanding of peptide diversity in Coleoptera, the structural atlas generated in this study (Table S3) is a valuable resource for exploring the evolutionary and functional diversity of small secreted peptides. Historically, the discovery of bioactive peptides has focused on a relatively limited number of well-characterized venomous organisms, whereas the enormous diversity of beetles has remained largely unexplored [8]. Our results demonstrate that transcriptome-wide structural mining can substantially broaden the repertoire of candidate peptide scaffolds available for future investigation.

An important implication of our findings is that structurally related peptides should not necessarily be expected to perform identical biological functions. Numerous studies have shown that highly similar peptide scaffolds may be repeatedly recruited during evolution for distinct physiological roles, including antimicrobial defense, immune modulation, hormone signaling, enzyme inhibition, and toxin activity [2,3,9]. Consequently, the structural similarity observed across many of our candidates likely reflects the retention or repeated emergence of robust molecular frameworks rather than strict conservation of biological function. This functional plasticity considerably expands the range of potential applications associated with the identified peptide families.

From a biotechnological perspective, compact disulfide-rich peptide scaffolds represent particularly attractive molecular templates due to their exceptional structural stability, resistance to proteolytic degradation, and capacity to tolerate sequence diversification while maintaining a conserved three-dimensional framework [4,5,6]. These properties have stimulated increasing interest in their application as starting points for therapeutic peptides, molecular recognition molecules, enzyme inhibitors, antimicrobial agents, and environmentally friendly bioinsecticides [3,12]. The diversity of structural architectures identified in the present study therefore considerably enlarges the repertoire of candidate scaffolds that may be explored for future biotechnology-driven optimization.

Equally important, the atlas provides an objective framework for selecting representative candidates for experimental validation. Rather than prioritizing peptides solely according to sequence similarity, the integration of structural clustering, AlphaFold 3 confidence estimates, Foldseek comparisons, and explainable machine learning (Figure 3, Figure 4 and Figure 5) enables the rational selection of structurally diverse representatives occupying distinct regions of the identified structural landscape. Such an approach maximizes the probability of capturing novel molecular functions while minimizing redundancy during downstream biochemical characterization. These results demonstrate that large-scale structure-guided transcriptome mining represents a powerful strategy for accelerating peptide discovery in taxonomically underexplored organisms.

### 3.5. Structural Bioinformatics Expands Functional Annotation Beyond Sequence Similarity

The rapid development of highly accurate protein structure prediction methods has substantially expanded the possibilities of computational biology. Until recently, large-scale structural characterization represented a major bottleneck for protein annotation, particularly in non-model organisms lacking experimentally determined structures. The emergence of AlphaFold 3 and related approaches has shifted this paradigm by enabling routine prediction of thousands of high-confidence protein structures, making the extraction of biological knowledge from these structural datasets the new computational challenge [11]. In this context, structural comparison algorithms such as Foldseek have become increasingly valuable because they enable the organization of large structural datasets according to three-dimensional similarity rather than primary sequence conservation.

Our workflow illustrates the advantages of integrating transcriptome mining, structure prediction, structural comparison, and interpretable machine learning into a unified framework for peptide discovery. We incorporated structural descriptors and Foldseek-derived relationships throughout the prioritization process, allowing candidate peptides to be evaluated within a broader structural context. This strategy enabled structurally related peptides originating from different beetle lineages to be grouped into recurrent protein architectures, while simultaneously preserving lineage-specific structural variants represented in the structural atlas (Figure 6; Figure S1). These observations reinforce that three-dimensional protein architecture has biologically meaningful information that complements conventional sequence-derived descriptors.

An important consequence of this integrative strategy is that structural information becomes useful not only for describing individual peptide models but also for organizing large repertoires of predicted proteins into biologically interpretable structural families. Because short extracellular peptides can exhibit extensive primary-sequence variation while retaining similar structural scaffolds, analyses based exclusively on sequence similarity may underestimate functional relationships among highly divergent candidates. By combining AlphaFold-derived models with Foldseek structural clustering, our framework identifies recurrent molecular architectures that remain detectable despite extensive sequence diversification, facilitating the recognition of representative scaffolds suitable for downstream experimental characterization.

Beyond the discovery of toxin-like peptides in Coleoptera, the computational strategy presented here provides a general framework for large-scale functional exploration of transcriptomic datasets from non-model organisms. As public repositories continue to expand and accurate structure prediction becomes increasingly accessible, workflows integrating transcriptomics, structural prediction, structural comparison, and explainable artificial intelligence are expected to play an increasingly important role in protein annotation, candidate prioritization, and the identification of previously unrecognized protein families. In this regard, the structural atlas generated in this study represents not only a catalog of predicted Coleoptera peptide structures but also a proof of concept demonstrating how structural bioinformatics can substantially enhance large-scale peptide discovery and functional hypothesis generation.

### 3.6. Limitations and Future Perspectives

Although the present study substantially expands the structural landscape of predicted bioactive peptides across Coleoptera, several limitations should be acknowledged. First, the identified candidates were inferred from transcriptomic data and computational analyses rather than experimentally validated at the protein level. Consequently, peptide expression, post-translational processing, structural stability under physiological conditions, and biological activities remain to be confirmed through complementary proteomic, biochemical, and functional assays. Nevertheless, recent advances in transcriptome-guided peptide discovery and high-accuracy structural prediction have demonstrated that integrative computational approaches display an efficient strategy for prioritizing candidates for downstream validation while substantially reducing experimental search space [11,12].

A second limitation concerns functional annotation. Similar protein architectures do not necessarily imply identical biological functions, and structurally related peptides may participate in diverse physiological processes, including immune defense, signaling, enzyme inhibition, and toxin activity [2,3,9]. Accordingly, the structural atlas presented here should be interpreted as a resource for hypothesis generation rather than definitive functional annotation. Future integration with transcriptomic expression profiles, comparative proteomics, receptor-binding studies, molecular dynamics simulations, and experimental functional assays will provide a more comprehensive understanding of the biological roles of these peptide families.

An additional limitation concerns candidates lacking predicted N-terminal signal peptides. Canonical secretion signals are expected for most extracellular bioactive peptides [15,16], transcriptome-based prediction may be affected by incomplete ORFs, assembly artifacts, or inaccurate delimitation of translation start sites [25]. Furthermore, some peptides may be secreted through non-classical pathways that are not detected by SignalP [15]. Consequently, these candidates should be interpreted cautiously, and their secretion mechanisms remain to be experimentally validated.

Despite these limitations, the framework developed in this study establishes a scalable strategy for large-scale exploration of peptide diversity in non-model organisms. As structural databases continue to expand and artificial intelligence-based prediction methods become increasingly accurate, comparative structural analyses will likely become a central component of peptide discovery pipelines. Extending this framework to additional insect orders and other underexplored metazoan lineages will enable increasingly comprehensive structural atlases, facilitating comparative evolutionary analyses and accelerating the identification of novel bioactive peptide scaffolds with potential applications in biotechnology and drug discovery [11,12,13].

## 4. Materials and Methods

To systematically identify and prioritize toxin-like peptides (TLPs) in Coleoptera, we developed an integrative computational pipeline combining sequence-based filtering, structural characterization, and machine learning-based classification. The workflow is summarized below.

### 4.1. Dataset Construction and Candidate Peptide Identification

We assembled/obtained a dataset comprising 88 publicly available transcriptomes spanning multiple beetle lineages. Species identity, transcriptome accession numbers, and the tissue, organ, body region, or developmental stage from which each biological sample was obtained were retrieved from the corresponding NCBI TSA/SRA records and associated BioSample metadata whenever available (Table S1). When the original public record did not specify the biological source in sufficient detail (NA—not available). Transcriptome assemblies were primarily obtained from the NCBI Transcriptome Shotgun Assembly (TSA) database, representing 70 unique TSA projects, with additional publicly available transcriptomic resources incorporated when required to maximize taxonomic coverage (Table S1). Raw RNA-seq datasets representing multiple Coleoptera species were assembled de novo using Trinity v2.15.2 [25]. The resulting transcriptomes were translated into putative open reading frames (ORFs) using TransDecoder v5.7.1, generating a comprehensive protein sequence dataset. To minimize redundancy and reduce sampling bias in downstream analyses, protein sequences were clustered using CD-HIT v4.8.1 (-c 0.9) [26], and representative sequences from each cluster were retained to construct a non-redundant dataset. Special attention was given to preserving short ORFs typically excluded during conventional annotation pipelines. Candidate proteins were screened using SignalP 5.0 [14] to identify canonical N-terminal secretion signals. Signal peptide prediction was subsequently incorporated as one of the prioritization criteria rather than as an absolute filtering step, allowing structurally promising candidates lacking a canonical signal peptide to be retained for downstream analyses [15]. Predicted signal peptide cleavage sites were subsequently used to delimit mature peptide regions.

The presence of a canonical N-terminal signal peptide is a hallmark of classically secreted bioactive peptides. However, candidates lacking a SignalP5 prediction were not automatically discarded. This decision was made because transcriptome-derived ORFs may represent incomplete transcripts, truncated precursor proteins, alternative translation products, or peptides secreted through non-classical pathways. Moreover, the primary objective of the pipeline was to maximize structural diversity during the discovery stage while allowing downstream prioritization to integrate multiple independent sources of evidence. Thus, the absence of a canonical signal peptide was treated as one feature contributing to candidate confidence rather than as an absolute exclusion criterion.

Predicted precursor sequences were further examined for features associated with peptide maturation, including propeptide regions and proteolytic cleavage motifs [27]. Mature peptide sequences were computationally extracted whenever possible, allowing downstream analyses to focus on biologically active regions rather than full-length precursor proteins. Candidate peptides were subsequently filtered according to physicochemical characteristics commonly associated with structurally constrained bioactive peptides, including peptide length, net charge, cysteine content and spacing, amphipathicity, hydrophobic moment (μH), amino acid composition, and other sequence-derived descriptors [28]. Net charge was calculated for each mature peptide at physiological pH (pH 7.0) by considering the ionization state of all charged amino acid side chains and terminal groups using Biopython/ProtParam. Following maturation inference, all sequence-derived physicochemical descriptors used in the mature-peptide analyses, including peptide length, molecular weight, net charge, isoelectric point, hydrophobicity, aromaticity, instability index, cysteine count, cysteine density, and cysteine spacing patterns, were recalculated exclusively from the inferred mature peptide sequences. Residues belonging to predicted signal peptides or propeptide regions were therefore excluded from these calculations. For cysteine-rich candidates, the maximum possible number of disulfide bonds was estimated from the number of cysteine residues present in the mature peptide sequence, whereas structure-based disulfide assignments were evaluated independently from the AlphaFold 3 models reconstructed using the corresponding mature sequences. These criteria were used to enrich the dataset while preserving structural and taxonomic diversity, allowing the inclusion of both canonical and potentially novel toxin-like peptide scaffolds.

All candidate sequences were screened against curated profile hidden Markov models using HMMER v3.4 [29] to identify conserved domains associated with well-characterized peptide families, including Kunitz inhibitors, Kazal domains, defensins, and cystine-knot peptides. Because short extracellular peptides frequently evolve rapidly and exhibit limited sequence conservation, both complete and partial HMM matches were considered during annotation. Importantly, sequences lacking significant HMM matches were retained for subsequent analyses, ensuring that highly divergent or previously undescribed peptide scaffolds were not excluded from the discovery pipeline.

The peptide sequences reported in this study were computationally inferred from publicly available transcriptomic datasets and therefore represent predicted peptide candidates. For this reason, they were not submitted as experimentally confirmed proteins to primary protein-sequence repositories. Instead, all predicted precursor and mature peptide sequences, together with their source accessions and associated metadata, were deposited in the permanent Zenodo archive and GitHub repository associated with this study.

### 4.2. Structure Prediction and Comparative Structural Analyses

Three-dimensional structural models were generated for all candidate mature peptides using AlphaFold 3 [11]. Model confidence was evaluated using the predicted Local Distance Difference Test (pLDDT), and only models with acceptable confidence scores were considered for downstream structural analyses. The resulting structures constituted the basis for all subsequent comparative analyses, enabling structural characterization independently of primary sequence similarity.

To comprehensively describe the structural properties of each peptide, multiple complementary descriptors were extracted from the predicted models. Secondary structure assignments were obtained using DSSP 4 [30], whereas solvent-accessible surface area (SASA) was calculated using FreeSASA v2.2.1 [31]. Additional structural descriptors, including radius of gyration, structural compactness, residue contact patterns, and other geometric measurements, were calculated with MDTraj v1.10.3 [32].

Structural relationships among candidate peptides were investigated through pairwise structural comparisons performed using Foldseek v.10-941cd33 [10], enabling the identification of structurally similar proteins even in the absence of detectable sequence homology. Foldseek similarity scores were subsequently used to identify recurrent structural architectures shared among peptides from distinct Coleoptera lineages. Unlike conventional sequence-based similarity searches, this approach enables the detection of structurally similar protein folds that remain recognizable despite extensive primary sequence variation. The structural descriptors extracted from AlphaFold 3 models together with Foldseek similarity metrics were integrated into downstream analyses to characterize the structural landscape of the identified peptide repertoire.

Representative structures from the mature-peptide clusters were additionally searched against external structural collections comprising experimentally determined structures from the Protein Data Bank (PDB) and predicted structures from the AlphaFold/Swiss-Prot collection. Structural relationships were evaluated using Foldseek with query-normalized TM-score, target-normalized TM-score, alignment coverage, and relative target length considered jointly to distinguish close scaffold-level counterparts from local geometric similarities. Candidates lacking close structural counterparts in the initial analysis were further evaluated using a high-sensitivity Foldseek search.

Mature peptide sequences were searched against the curated UniProtKB/Swiss-Prot protein database using BLASTP v.2.12.0+. For each representative structural cluster, the highest-scoring informative hit was retained together with its UniProt accession number, sequence identity, query coverage, *E*-value, bit score, and protein description. Because short peptides can generate local similarities to unrelated proteins, BLAST v.2.12.0+ results were interpreted as supporting sequence-similarity evidence rather than definitive functional assignments.

### 4.3. Structure-Guided Machine Learning Prioritization

We developed an interpretable machine learning framework to prioritize candidates for downstream analyses integrating sequence-derived, physicochemical, and structural descriptors into a unified predictive model. Unlike conventional sequence-based peptide discovery pipelines, our strategy incorporated information extracted from AlphaFold 3 models and comparative structural analyses, allowing structurally similar peptides to be identified even in the presence of extensive sequence divergence. Because experimentally validated negative examples of toxin-like peptides are largely unavailable, candidate prioritization was formulated as a Positive-Unlabeled (PU) learning problem. This strategy enables robust classification when only confirmed positive examples and unlabeled sequences are available, avoiding biases associated with artificially generated negative datasets. The training dataset consisted of experimentally characterized toxin-like peptides representing diverse peptide families, whereas all remaining candidates were treated as unlabeled observations.

The positive training dataset was derived from the curated reference peptide collection previously developed by our group for large-scale structural analyses of venom- and toxin-derived peptides [13]. This reference dataset integrates experimentally characterized bioactive peptides obtained from manually curated public resources, including UniProtKB/Swiss-Prot (ToxProt subset), NCBI Protein, and peer-reviewed literature. Only experimentally supported or expert-curated entries were retained following quality-control procedures that included redundancy reduction, removal of incomplete or fragmented sequences, and sequence standardization. For the present study, this reference collection was used exclusively as the positive class for PU-learning, whereas the Coleoptera-derived candidates identified here constituted the unlabeled dataset to be prioritized. The reference dataset comprises representatives of multiple experimentally characterized toxin-like peptide families, including defensins, Kunitz-type inhibitors, Kazal-domain peptides, cystine-knot (ICK) peptides, conotoxin-like peptides, and other structurally characterized bioactive peptide scaffolds.

Each candidate was represented by a comprehensive feature set combining sequence-derived descriptors, physicochemical properties, structural metrics extracted from predicted three-dimensional models, and Foldseek similarity scores. These features included peptide length, molecular weight, amino acid composition, net charge, isoelectric point, hydrophobicity, aliphatic index, cysteine content, secondary-structure composition, solvent-accessible surface area, radius of gyration, structural compactness, pLDDT confidence scores, and comparative structural similarity metrics. This integrated representation enabled simultaneous evaluation of biochemical and structural characteristics during candidate prioritization.

To improve robustness and reduce stochastic variation, the PU-learning framework was implemented as an ensemble of five independently trained models initialized with different random seeds. Each model was trained using the same positive reference dataset and identical feature set, while random initialization affected only the stochastic components of the learning process. Candidate prioritization was based on the mean PU score across all ensemble members, whereas ranking stability was quantified according to the frequency with which each candidate appeared among the top-ranked predictions across independent runs. This ensemble strategy reduced seed-dependent variability and provided an additional measure of prediction reproducibility.

Classification models were implemented in Python 3.10 using scikit-learn [24]. Hyperparameter optimization and model selection were performed using repeated cross-validation, and predictive performance was evaluated through Receiver Operating Characteristic (ROC) and Precision–Recall (PR) curves. Because peptide discovery represents a highly imbalanced classification problem, PR curves were considered particularly informative for assessing classifier performance [23], while complementary probabilistic performance measures followed the recommendations of Gajda and Chlebus [20].

Model predictions were examined using SHapley Additive exPlanations (SHAP), which quantify the contribution of individual features to each prediction and improve biological interpretability [21,33]. The resulting prioritization scores were not interpreted as definitive functional annotations but rather as confidence estimates integrating multiple independent sources of evidence. These scores were subsequently combined with structural clustering analyses to guide the selection of representative candidates for the structural atlas and future experimental validation.

Taxonomic information associated with each transcriptome was standardized using the taxize v.0.10.0 package [34] in R v.4.1.2 [35]. Candidate peptides were subsequently grouped according to their taxonomic classification to investigate the distribution of predicted bioactive peptide scaffolds across Coleoptera lineages and to identify peptide families exhibiting lineage-specific enrichment. Associations between peptide occurrence and taxonomic groups were evaluated using Fisher’s Exact Test [36]. To account for multiple hypothesis testing, *p*-values were adjusted using the Benjamini–Hochberg false discovery rate (FDR) procedure [37], and corrected values below 0.05 were considered statistically significant.

All statistical analyses and data manipulation were performed in R [35], primarily using the dplyr [38] and tidyr [39] packages. Summary statistics were calculated for sequence-derived, physicochemical, structural, and machine learning-derived descriptors, and graphical visualizations were generated to support comparative analyses presented throughout the study.

### 4.4. Structural Atlas Construction

The final structural atlas was generated by integrating sequence-derived descriptors, physicochemical properties, predicted three-dimensional structures, comparative structural analyses, and machine learning prioritization into a unified dataset. Each candidate peptide was represented by its mature amino acid sequence, structural model, physicochemical profile, structural descriptors, Foldseek similarity metrics, and prioritization score, providing a comprehensive overview of its predicted structural and biochemical characteristics.

Peptides were organized according to structural similarity to facilitate comparative analyses. Foldseek-derived structural comparisons enabled the identification of recurrent protein architectures shared among candidate peptides, allowing structurally related molecules to be grouped even when sequence conservation was minimal. Representative structures from each structural group were selected to summarize the diversity of the identified peptide repertoire while preserving the major architectural features observed across the dataset. Machine learning prioritization scores were subsequently integrated with structural information to support the selection of high-confidence candidates for downstream analyses.

All scripts used for feature extraction, structural analysis, Foldseek clustering, PU-learning, ensemble generation, figure production, and statistical analyses are publicly available through the GitHub repository together with the complete feature matrices, configuration files, intermediate datasets, representative structural models, and processed outputs required to reproduce the computational workflow.

## 5. Conclusions

Here, we present the first large-scale structural atlas of predicted toxin-like peptide scaffolds across Coleoptera, revealing that beetle transcriptomes harbor a substantially broader repertoire of structurally constrained bioactive peptides than previously recognized. Despite extensive primary-sequence divergence, many candidates were organized into a comparatively limited set of recurrent protein architectures, indicating that structural similarity represents a major organizing feature of peptide diversity identified in this insect order. These findings expand current knowledge of peptide evolution and demonstrate that protein structure offers a more informative framework than primary sequence alone for exploring hidden bioactive peptide diversity.

By integrating transcriptome mining, peptide maturation prediction, structural modeling, comparative structural analyses, and interpretable machine learning, we establish a scalable framework for structure-guided peptide discovery in underexplored taxa. Beyond providing a comprehensive catalog of high-confidence candidates, the structural atlas generated here represents a valuable resource for future evolutionary, structural, biochemical, and biotechnological investigations. Collectively, our results reinforce the growing importance of structure-based approaches for uncovering novel bioactive peptide scaffolds and provide a foundation for accelerating peptide discovery across the vast unexplored diversity of animal life.

## Figures and Tables

**Table 1:** Summary of candidate discovery and maturation filtering in Coleoptera transcriptomes.

Step	N	(%)
Initial candidates	291	100.0
Predicted secreted (Signal peptide)	155	53.3
Valid mature peptide length (8–80 aa)	273	93.8
Out-of-range mature peptides	18	6.2
Candidates with dibasic cleavage motifs	127	43.6

**Table 2:** Sequence and physicochemical properties of the 76 high-confidence mature Coleoptera toxin-like peptide candidates retained after the revised maturation and filtering workflow. Net charge was calculated at pH 7.0. Cysteine count, cysteine density, and the maximum possible number of disulfide bonds therefore refer exclusively to the mature peptide sequence.

No.	Candidate_ID	Species	Mature_Peptide_Sequence	Mature_Length_aa	Molecular_Weight_Da	Isoelectric_Point	Net_Charge_pH7	Gravy	Cysteine_Count	Cysteine_Density	Maximum_Possible_Disulfides
1	allokotarsa_sp_2	*Allokotarsa* sp.	D VEAERICRLPPEDTSSGISCLALIPKYTYSAAEEKCVPYQYGGCNGTENLFSTEDECKKKCDGVKTH	68	7493. 31	4.81	−4.19	−0.63	6	0.09	3
2	allokotarsa_sp_3	*Allokotarsa* sp.	ITPDTSDYICKLPAKKGFCRAYIQRYYYDVKAKKCKEFIYTGCGGNENSFDSLQDCTAKCKNVK	64	7333.36	8.93	4.70	−0.67	6	0.09	3
3	alurnus_ornatus_3	*Alurnus ornatus*	KPCLKDCGNDYKPVCGGDGSGKGNKSFGSKCVLENYNCEQGINWSIQSQGECPGGGGVRLQ	61	6369.04	7.75	0.70	−0.77	6	0.10	3
4	anomala_sp_2	*Anomala* sp.	KCVKACTDDYTPVCGGDDAGKKISFGNECVLANYNCENNANLKLVSKGECPGSQGVRLS	59	6185.91	6.28	−0.29	−0.41	6	0.10	3
5	aphodius_haemorrhoidalis_1	*Aphodius haemorrhoidalis*	TEKKECLKVCPDDYTPVCGTDADGKKKLSFGNECVMNNYNCEQGANLHVLSKGECPGGQGVRLS	64	6880.71	5.66	−1.56	−0.67	6	0.09	3
6	aphodius_scrutator_2	*Aphodius scrutator*	TQTIACTANCVSGCFCNPGYLHNSKGKCVKEANCS	35	3653.15	8.28	1.42	−0.13	6	0.17	3
7	bolboceras_sp_1	*Bolboceras* sp.	SDLRIVREGVC	11	1246.44	5.79	−0.55	0.18	1	0.09	0
8	brassicogethes_aeneus_2	*Brassicogethes aeneus*	VCEKEMFWGCRATPNNFRTLEECLTVAKPICTQ	33	3819.43	6.24	−0.30	−0.21	4	0.12	2
9	byturus_ochraceus_1	*Byturus ochraceus*	PKPQICTAQCVTGCFCLRGYIRNGITGECVRPNQCPPDSAYRPGK	45	4899.66	8.96	3.90	−0.45	6	0.13	3
10	catharsius_molossus_1	*Catharsius molossus*	FCNPVLGQYCVPGEAFMRDCNTCICSLDGRDAACTLKACFYG	42	4557.26	4.68	−1.31	0.29	7	0.17	3
11	catharsius_molossus_2	*Catharsius molossus*	IDCNGCRCVNGDWACTRKICHRSTQREYCVPGEPFMQDCNTCICAADGRNAACTMKACLYR	61	6827.84	8.08	1.74	−0.35	11	0.18	5
12	ceutorhynchus_napi_1	*Ceutorhynchus napi*	CERTIYGGCRATRNNFATMDICQEVAGTICSQS	33	3600.03	6.15	−0.27	−0.21	4	0.12	2
13	ceutorhynchus_napi_3	*Ceutorhynchus napi*	VCEKEMFWGCRATPNNFRTLEECLTVAKPICTQ	33	3819.43	6.24	−0.30	−0.21	4	0.12	2
14	copris_lunaris_3	*Copris lunaris*	KCLKECPDDYTPVCGTDDGGKKLSFGNECVLNNYNCEQSANLRVLSKGECPGGQGIRLS	59	6326.05	5.14	−1.29	−0.62	6	0.10	3
15	cucujus_clavipes_1	*Cucujus clavipes*	CVKFVYGGCNGNNNNFKTLKECEAIALPICAES	33	3551.06	6.2	−0.27	0.02	4	0.12	2
16	dichotomius_satanas_2	*Dichotomius satanas*	IDCNDCRCINGNWGCTKKACGPAKRETTCNPGETFKRGCNTCVCSPDGKNAACTLKAC	58	6133.02	8.55	3.65	−0.55	11	0.19	5
17	dichotomius_satanas_4	*Dichotomius satanas*	LKWDNNLANAAQNIANSCEFAHKSVSDGK	29	3146.41	6.74	−0.16	−0.67	1	0.03	0
18	diphucephala_sp_2	*Diphucephala* sp.	TCGPACHPTCANPNVSTVSCPKPCISGCFCKADFLTNSKGKCVPKNQCS	49	5048.87	8.65	3.39	−0.14	9	0.18	4
19	diphucephala_sp_3	*Diphucephala* sp.	TCGPACHPTCANPNVSTVSCPKPCISGCFCKANFLTNSKGKCVPKNQCS	49	5047.88	8.84	4.39	−0.14	9	0.18	4
20	elateroides_flabellicornis_3	*Elateroides flabellicornis*	CQVQIDMNELMAKREQILGRRGKDY	25	2995.46	8.17	0.75	−0.9	1	0.04	0
21	eophileurus_sp_2	*Eophileurus* sp.	STDDNCTKECRTGCVCKPGYIYKGPRCVLPQDC	33	3654.18	7.51	0.40	−0.65	6	0.18	3
22	euoniticellus_fulvus_1	*Euoniticellus fulvus*	PRACAFNCIPGCFCKSGYLYNAKGNCVKESQC	32	3476.04	8.72	2.90	−0.12	6	0.19	3
23	euwallacea_fornicatus_1	*Euwallacea fornicatus*	CKQFVFGGCSKRITNNFPSKKTCMDTCSDVW	31	3532.08	8.89	2.72	−0.39	4	0.13	2
24	gaco00000000_1	*Priacma serrata*	NMPDICKLPLAPTDGRMCLAYFERYYYNQSQQRCVKFIYGGCRGNTNNFYSLEQCQETCESMDDNLL	67	7864.82	4.77	−2.29	−0.63	6	0.09	3
25	gbcx00000000_1	*Dastarcus helophoroides*	HQEGPQCRAYIPKFSWDNNSKQCKEVIYGGCHGTNNLFNTFQECERTALPICKEV	55	6334.06	6.89	−0.11	−0.78	5	0.09	2
26	gdlg00000000_1	*Byturus ochraceus*	PKPQICTAQCVTGCFCLRGYIRNGITGECVRPNQCPPDSAYRPGK	45	4899.66	8.96	3.90	−0.45	6	0.13	3
27	gdmj00000000_1	*Hydroscapha redfordi*	ATCDLLSFFNVNHSACALHCIARGNRGGYCNGQKVCVCRN	40	4316.93	8.69	2.91	0.07	6	0.15	3
28	gdmq00000000_1	*Sitophilus zeamais*	QCVPAVYGGCRRTNNNFPTLEQCELSARSVCISNAI	36	3915.42	7.88	0.72	−0.09	4	0.11	2
29	gdnw00000000_1	*Cucujus clavipes*	CVKFVYGGCNGNNNNFKTLKECEAIALPICAES	33	3551.06	6.2	−0.27	0.02	4	0.12	2
30	gdof00000000_2	*Alurnus ornatus*	KPCLKDCGNDYKPVCGGDGSGKGNKSFGSKCVLENYNCEQGINWSIQSQGECPGGGGVRLQ	61	6369.04	7.75	0.70	−0.77	6	0.10	3
31	geotrupes_spiniger_2	*Geotrupes spiniger*	DGVCVPPNKCGK	12	1216.43	8.04	0.74	−0.45	2	0.17	1
32	gfuh00000000_2	*Amphizoa lecontei*	FNQMRGIELRIVKEDRCSNEIANA	24	2807.17	6.25	−0.24	−0.60	1	0.04	0
33	ghma00000000_1	*Dichotomius satanas*	IDCNDCRCINGNWGCTKKACGPAKRATTCNPGETFKQGCNTCVCSPDGKNAACTLKAC	58	6046.92	8.55	3.65	−0.45	11	0.19	5
34	ghma00000000_3	*Dichotomius satanas*	GEGACHGDSGGPLVEGEHQVGVVSWGIPCAKGKPDVFTRVYRYLDWVVNYMKD	53	5780.44	5.49	−2.07	−0.30	2	0.04	1
35	ghmd00000000_4	*Onthophagus curvicornis*	NLRCALKCSTGCYCKPGYLRNYNGQCVKVKDC	32	3602.24	9.08	4.69	−0.43	6	0.19	3
36	ghxo00000000_6	*Acalymma vittatum*	ATCDLLSGTGANHSACAAHCLLRGNRGGYCNSKAVCVCRN	40	4068.61	8.69	2.91	0.02	6	0.15	3
37	gjbk00000000_1	*Catharsius molossus*	FCNPVLGQYCVPGEAFMRDCNTCICSLDGRDAACTLKACFYG	42	4557.26	4.68	−1.31	0.29	7	0.17	3
38	gjbk00000000_2	*Catharsius molossus*	IDCNGCRCVNGDWACTRKICHRSTQREYCVPGEPFMQDCNTCICAADGRNAACTMKACLYR	61	6827.84	8.08	1.74	−0.35	11	0.18	5
39	gjvn00000000_1	*Brassicogethes aeneus*	DCEKEMFWGCRATPNNFRTLEECLTVAKPICTQ	33	3835.39	5.01	−1.27	−0.44	4	0.12	2
40	gjvo00000000_1	*Ceutorhynchus napi*	KDCLAPHTEPGATCKAHLIRYHWNQTLKACEEVIYGGCRATRNNFKTMEECNKVAGSICNKKCKK	65	7342.47	9.08	5.96	−0.71	7	0.11	3
41	gkjd00000000_2	*Serica brunnea*	IGPDTSEYICKLPLKKGVCRAYIQRYYYDVKARKCKEFVYTGCAGNENSFDTIQDCTAKCKHVK	64	7362.45	8.95	4.79	−0.58	6	0.09	3
42	gkjd00000000_3	*Serica brunnea*	DEAESICRQPKRVGRCRASFPKFYYNPEAKECQKFVYGGCDGNQNSFNTKADCTAKCGKLK	61	6887.73	9.00	4.70	−1.00	6	0.10	3
43	gkjh00000000_2	*Anomala* sp.	KCVKACTDDYTPVCGGDDAGKKISFGNECVLANYNCENNANLKLVSKGECPGSQGVRLS	59	6185.91	6.28	−0.29	−0.41	6	0.10	3
44	gkjj00000000_1	*Astenopholis* sp.	GCVCKEGYARILRYGICVANFSCNQYIP	28	3141.67	8.51	1.72	0.27	4	0.14	2
45	gkjr00000000_1	*Ocypus brunnipes*	HCVKTRETTCKPGETFKMDCNTCTCSADGKNAACTYKLCY	40	4427.07	8.24	1.78	−0.58	7	0.17	3
46	gkke00000000_1	*Diphucephala* sp.	TCGPACHPTCANPNVSTVSCPKPCISGCFCKADFLTNSKGKCVPKNQCS	49	5048.87	8.65	3.39	−0.14	9	0.18	4
47	gkke00000000_2	*Diphucephala* sp.	TCGPACHPTCANPNVSTVSCPKPCISGCFCKADFLTNSKGKCVPKNQCS	49	5048.87	8.65	3.39	−0.14	9	0.18	4
48	hbua00000000_1	*Diabrotica virgifera*	CNDDCYCIPGYARISTNGRCIPIDKCPKKCKTIT	34	3794.45	8.64	2.70	−0.41	6	0.18	3
49	heteronyx_sp_3	*Heteronyx* sp.	KTCAKECSDDYTPICGGDNQGKKLSFGNECVMNNYNCEQNANLRVLSKGECPGSQGVRLS	60	6476.17	6.30	−0.29	−0.77	6	0.1	3
50	hydroscapha_redfordi_1	*Hydroscapha redfordi*	ATCDLLSFFNVNHSACALHCIARGNRGGYCNGQKVCVCRN	40	4316.93	8.69	2.91	0.07	6	0.15	3
51	hygrobia_hermanni_1	*Hygrobia hermanni*	STGVCVLKSKC	11	1124.38	8.88	1.44	0.61	2	0.18	1
52	iclg00000000_1	*Zophobas atratus*	GFLKNGQGQCVPPHAC	16	1655.9	8.07	0.83	−0.27	2	0.12	1
53	lepiserica_sp_1	*Lepiserica* sp.	GFLLNSKGKCVRDNQC	16	1782.05	8.90	1.74	−0.52	2	0.12	1
54	lepiserica_sp_4	*Lepiserica* sp.	ITPDTSEYICKLPLRKGVCRAYIQRYYYDVNARKCKEFVYTGCAGNENSFDTLQDCTAKCKNIK	64	7411.44	8.79	3.70	−0.60	6	0.09	3
55	lepiserica_sp_5	*Lepiserica* sp.	VGRCKAYFPKFYYNPEAKECQEFVYGGCDGNQNSFNTQADCMAKCGKIK	49	5553.25	8.34	1.68	−0.74	5	0.10	2
56	maechidius_sp_1	*Maechidius* sp.	CKNFIYGGCQGNGNNFPTISECEAKCSGVL	30	3154.53	6.11	−0.28	−0.17	4	0.13	2
57	microcara_testacea_1	*Microcara testacea*	GQEQNYCKAYIKSYWFNIATKKCEYYIYGGCGATENRFLNAKDCLETCSKYLQ	53	6222.99	8.32	1.71	−0.66	5	0.09	2
58	migdolus_fryanus_1	*Migdolus fryanus*	SDCDQPLTEPGIQCYGYFIRYYWSNTAQKCIQGVYGGCRATNNNFKTLKECEEIAGSVCKKKHD	64	7275.11	7.53	0.49	−0.72	6	0.09	3
59	migdolus_fryanus_4	*Migdolus fryanus*	FSSSDCSLNVHTPGPYGQICAAYFPVYKWSNSEQKCVKAVYGGCRATNNNFESDEECNKIAKPICANN	68	7471.23	6.72	−0.21	−0.53	6	0.09	3
60	ocypus_brunnipes_1	*Ocypus brunnipes*	HCVKTRETTCKPGETFKMDCNTCTCSADGKNAACTYKLCY	40	4427.07	8.24	1.78	−0.58	7	0.17	3
61	orphnus_sp_1	*Orphnus* sp.	GVCRAYLERYYYDVKTGNCKLFVYGGCSGNANSFESIEECVTRCKSIN	48	5412.05	7.79	0.71	−0.31	5	0.10	2
62	orphnus_sp_2	*Orphnus* sp.	GKCVPKGSC	9	878.07	8.90	1.74	−0.2	2	0.22	1
63	phyllotocus_sp_3	*Phyllotocus* sp.	VNTNSNNICQLPVKKGICRAYIERYYHDVKSRNCKLFVYSGCGGNENNFETKSECKAKCSNVQ	63	7168.05	9.00	4.76	−0.75	6	0.09	3
64	psylliodes_chrysocephalus_1	*Psylliodes chrysocephalus*	VCEKEMFWGCRATPNNFRTLEECLTVAKPICTQ	33	3819.43	6.24	−0.30	−0.21	4	0.12	2
65	psylliodes_chrysocephalus_2	*Psylliodes chrysocephalus*	KDCLAPHTEPGATCKAHLIRYHWNQTLKACEEVIYGGCRATRNNFKTMEECNKVAGSICNKKCKK	65	7342.47	9.08	5.96	−0.71	7	0.11	3
66	scitalia_sp_1	*Scitalia* sp.	FFYDLETKTCDRFIYGGCKEGDNNFLYLWECQKMCKGIVILHD	43	5157.9	5.09	−2.18	−0.23	4	0.09	2
67	serica_brunnea_1	*Serica brunnea*	DEAESICRQPKRVGRCRASFPKFYYNPEAKECQKFVYGGCDGNQNSFNTKADCTAKCGKLK	61	6887.73	9.00	4.70	−0.10	6	0.10	3
68	sitophilus_oryzae_2	*Sitophilus oryzae*	QCVPAVYGGCRRTNNNFPTLEQCELAARSVCVSNAI	36	3885.39	7.88	0.72	−0.02	4	0.11	2
69	sitophilus_zeamais_2	*Sitophilus zeamais*	QCVPAVYGGCRRTNNNFPTLEQCELSARSVCVSNAI	36	3901.39	7.88	0.72	−0.10	4	0.11	2
70	spercheus_emarginatus_2	*Spercheus emarginatus*	CVLLEQCTK	9	1036.27	5.99	−0.26	0.58	2	0.22	1
71	srr1562204_2	*Migdolus fryanus*	FSSSDCSLDVHTPGPYGQICAAYFPVYKWSNSEQKCVKAVYGGCRATNNNFESDEECNKIAKPICANN	68	7472.21	5.64	−1.21	−0.53	6	0.09	3
72	srr1562204_4	*Migdolus fryanus*	SDCDQPLTEPGIQCYGYFIRYYWSNTAQKCIQGVYGGCRATNNNFKTLKECEEIAGSVCKKKHD	64	7275.11	7.53	0.49	−0.72	6	0.09	3
73	srr1562205_1	*Migdolus fryanus*	SDCDQPLTEPGIQCYGYFIRYYWSNTAQKCIQGVYGGCRATNNNFKTLKECEEIAGSVCKKKHD	64	7275.11	7.53	0.49	−0.72	6	0.09	3
74	srr1562205_4	*Migdolus fryanus*	FSSSDCSLNVHTPGPYGQICAAYFPVYKWSNSEQKCVKAVYGGCRATNNNFESDEECNKIAKPICANN	68	7471.23	6.72	−0.21	−0.53	6	0.09	3
75	srr19974962_1	*Sitophilus oryzae*	QCVPAVYGGCRRTNNNFPTLEQCELSARSVCVSNAI	36	3901.39	7.88	0.72	−0.10	4	0.11	2
76	srr2083703_1	*Ocypus brunnipes*	HCVKTRETTCKPGETFKMDCNTCTCSADGKNAACTYKLCY	40	4427.07	8.24	1.78	−0.58	7	0.17	3

## Data Availability

The processed datasets, predicted structural models, analysis scripts, structural-clustering results, and source data supporting this study are available through the GitHub repository (https://github.com/BBMDO/coleotoxmin accessed on 16 August 2026) and the associated Zenodo record (DOI 10.5281/zenodo.21386392). The deposited material includes the predicted mature peptide sequences, transcriptome metadata and source accession numbers, processed feature matrices, AlphaFold 3 structural models, Foldseek clustering results, BLAST similarity results, functional annotations, machine-learning inputs and outputs, figure source data, and all scripts required to reproduce the analyses presented in the revised manuscript. The curated positive reference dataset used for PU-learning is also available in the project repository together with the feature matrix used for model training. Raw transcriptomic datasets were obtained from public repositories, and their accession numbers are provided in Table S1.

## Supplementary Materials

The following supporting information can be downloaded at: https://www.mdpi.com/article/10.3390/ijms27167489/s1.
