# Supplementary material for: Structure-Guided Discovery Reveals Recurrent Bioactive Peptide Architectures Across Coleoptera
Source: Int J Mol Sci. 2026 Aug 21;27(16):7489. doi: 10.3390/ijms27167489 (PMC13513286; doi:10.3390/ijms27167489)
Supplement: Supplementary file 1 [file ijms-27-07489-s001.zip › ijms-4477523-supplementary figure.pdf]

Structure-guided discovery reveals recurrent bioactive peptide architectures across Coleoptera

Thaís Caroline Gonçalves, João Alfredo Teodoro, and Danilo T. Amaral*

Centro de Ciências Naturais e Humanas, Universidade Federal do ABC (UFABC), Santo André, São Paulo, Brazil.

*Corresponding author, Avenida dos Estados, 5001, bloco A, room 504-3. ZIP CODE 09210-580, Santo André, Brazil. email: danilo.trabuco@ufabc.edu.br

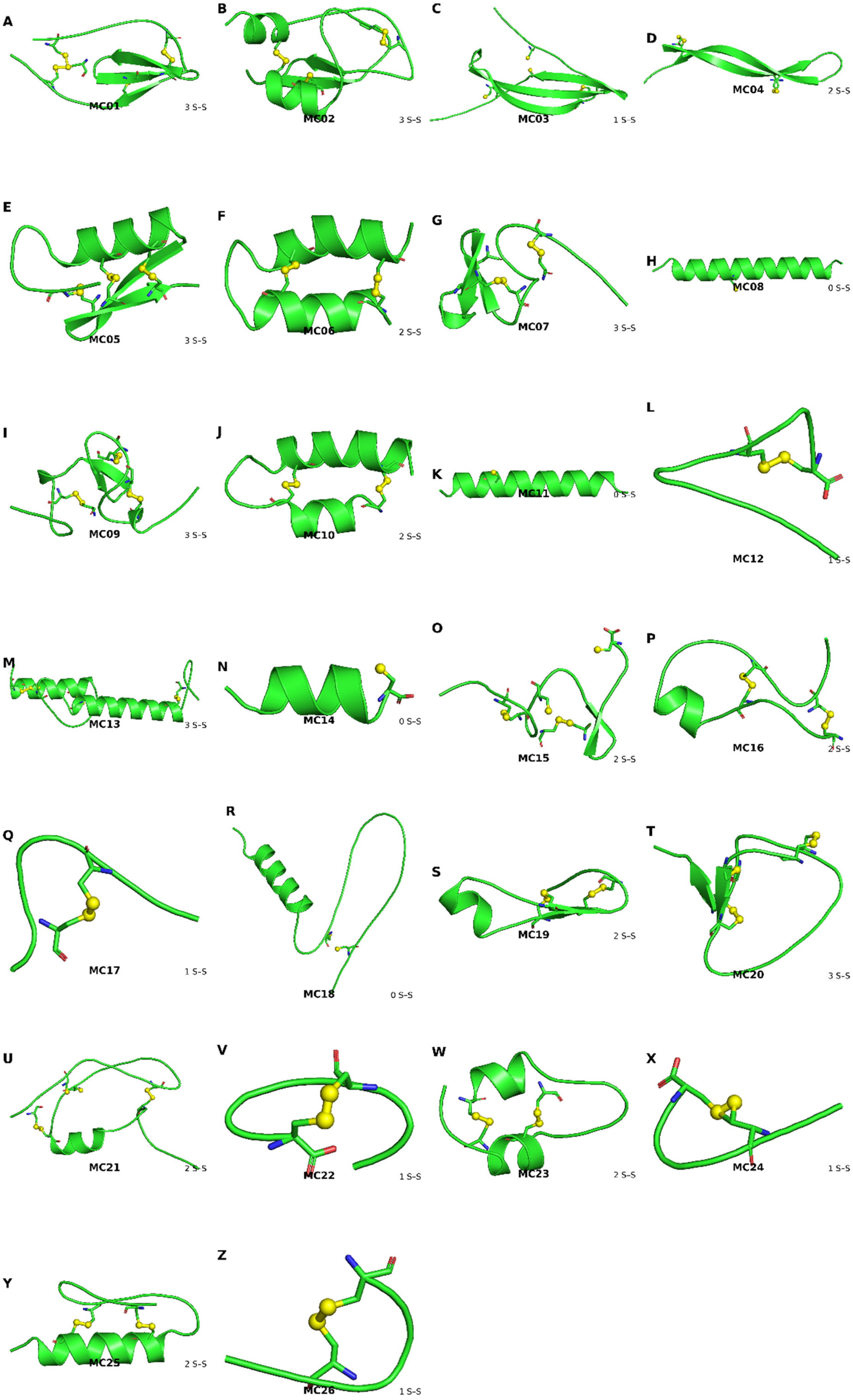
A
MC01
3 S-S
B
MC02
3 S-S
C
MC03
1 S-S
D
MC04
2 S-S
E
MC05
3 S-S
F
MC06
2 S-S
G
MC07
3 S-S
H
MC08
0 S-S
I
MC09
3 S-S
J
MC10
2 S-S
K
MC11
0 S-S
L
MC12
1 S-S
M
MC13
3 S-S
N
MC14
0 S-S
O
MC15
2 S-S
P
MC16
2 S-S
Q
MC17
1 S-S
R
MC18
0 S-S
S
MC19
2 S-S
T
MC20
3 S-S
U
MC21
2 S-S
V
MC22
1 S-S
W
MC23
2 S-S
X
MC24
1 S-S
Y
MC25
2 S-S
Z
MC26
1 S-S

Figure S1. Representative AlphaFold 3 models of all 26 structural clusters obtained from the 76 high-confidence mature peptide sequences. Predicted disulfide bonds between cysteine residues are explicitly displayed in the structural models. For each cluster, the cluster identifier, number of mature peptide members (*n*), mean pLDDT, and external structural-similarity classification are indicated. MC08 (*) remained without a close structural counterpart after sensitive searches against PDB and AlphaFold/Swiss-Prot. † indicates mean pLDDT < 60 and therefore lower confidence in the predicted topology.
